# Women Are More Susceptible to Caries but Individuals Born with Clefts Are Not

**DOI:** 10.1155/2011/454532

**Published:** 2011-06-08

**Authors:** Aditi Jindal, Michelle McMeans, Somnya Narayanan, Erin K. Rose, Shilpa Jain, Mary L. Marazita, Renato Menezes, Ariadne Letra, Flavia M. Carvalho, Carla A. Brandon, Judith M. Resick, Juan C. Mereb, Fernando A. Poletta, Jorge S. Lopez-Camelo, Eduardo E. Castilla, Iêda M. Orioli, Alexandre R. Vieira

**Affiliations:** ^1^Departments of Oral Biology, School of Dental Medicine, University of Pittsburgh, Pittsburgh, PA 15261, USA; ^2^Center for Dental and Craniofacial Genetics, School of Dental Medicine, University of Pittsburgh, Pittsburgh, PA 15261, USA; ^3^Division of Pediatric Hematology-Oncology, Children's Hospital of Pittsburgh, Pittsburgh, PA 15224, USA; ^4^Department of Genetics, Center of Health Sciences, Institute of Biology, Federal University of Rio de Janeiro, Rio de Janeiro, RJ, Brazil; ^5^(ECLAMC) Latin American Collaborative Study of Congenital Malformations at Hospital de Area El Bolsón, Río Negro, Argentina; ^6^(ECLAMC at CEMIC) Center for Medical Education and Clinical Research, Buenos Aires, Argentina; ^7^(CONICET) National Research Council of Argentina, Argentina; ^8^(INAGEMP-CNPq)National Institute of Population Medical Genetics, Brazil; ^9^(ECLAMC at INAGEMP-CNPq) National Institute of Population Medical Genetics in CEMIC, Buenos Aires, Argentina; ^10^(ECLAMC at IMBICE )Multidisciplinary Institute of Cellular Biology, La Plata, Argentina; ^11^Pediatric Dentistry, School of Dental Medicine, University of Pittsburgh, Pittsburgh, PA 15261, USA

## Abstract

The identification of individuals at a higher risk of developing caries is of great interest. Isolated forms of cleft lip and palate are among the most common craniofacial congenital anomalies in humans. Historically, several reports suggest that individuals born with clefts have a higher risk for caries. Caries continues to be the most common infectious noncontagious disease worldwide and a great burden to any health system. The identification of individuals of higher susceptibility to caries is of great interest. In this paper, we assessed caries experience of 1,593 individuals from three distinct populations. The study included individuals born with clefts, their unaffected relatives, and unrelated unaffected controls that were recruited from areas with similar cultural pressures and limited access to dental care. DMFT/dmft scores were obtained, and caries experience rates were compared among the three groups in each geographic area. Individuals born with clefts did not present higher caries experience in comparison to their unaffected relatives or unrelated unaffected controls. Women tend to present higher caries rates in comparison to men. Our work provides strong evidence that individuals born with clefts are not at higher risk to caries; however, women tend to have more severe caries experience.

## 1. Introduction

The identification of individuals at a higher risk of developing caries is of great interest. Among the suggested high risk individuals are the ones born with cleft lip and palate. Most reports suggest individuals born with clefts have a higher risk of caries [1–19]. There are studies however that did not find any difference in caries experience between individuals born with clefts and unaffected controls [20–26]. It has been proposed that the higher incidence of caries in the cleft populations reported in many studies are likely due to poor study design [27], diet rich in sugars [28, 29], poor oral hygiene [30–32], lack of motivation to perform regular preventive dental home care for the children [28, 29], low fluoride exposure [20, 24], early infection by *Streptococcus mutans* and lactobacilli [33, 34], teeth misalignment [35], and high prevalence of dental erosion [24]. The most recent meta-analysis of data on the frequency of caries in individuals born with clefts was unable to conclude that individuals born with these defects have higher frequency of caries [27]. Since there is still no consensus in the literature regarding this matter, we decided to analyze caries experience data in three independent populations of lower socioeconomic status and with limited access to dental care to test the hypothesis that individuals born with clefts have a higher caries susceptibility. Our analysis suggests individuals born with clefts are not more susceptible to caries.

## 2. Subjects and Methods

All subjects were recruited as part of studies aiming to identify genetic factors contributing to clefts. Individuals born with isolated forms of cleft lip with or without cleft palate, their relatives, and unrelated unaffected individuals were invited to participate. The University of Pittsburgh Institutional Review Board, as well as the corresponding appropriate boards in each study site approved this project. All subjects provided written informed consent before participating in this study. Age appropriate documents were used for children between ages 8 and 13 years. Parents provided consent to children seven years of age and under.

In this study, caries experience data of 1,593 subjects were analyzed. Six hundred twenty-eight subjects were recruited from the Cebu province in the Philippines. Five hundred and ninety-four subjects were recruited from Guatemala (cities of Pueblo Nuevo Tiquisate, Retalhuleu, and Santa Cruz del Quiché). Finally, 371 subjects were recruited from the Patagonian region of Argentina (cities of ChoeleChoel, El Bolsón, Esquel, General Roca, IngenieroJacobacci, Maquinchao, El Maitén, Rio Colorado, San Antonio Oeste, San Carlos de Bariloche, Sierra Grande, and Valcheta). In all these sites, individuals were from modest socioeconomic backgrounds, with limited access to dental care, and similar regional cultural influences.

Caries experience was recorded by the DMFT index (Decayed, Missing due to caries, Filled Teeth) as recommended by the World Health Organization [36]. One of the authors (A. R. V.) was responsible for calibration of all the examiners. In addition to A. R. V., two individuals carried out the clinical examination, after being calibrated, in the Philippines, two individuals in Guatemala, and one individual in Argentina. ANOVA, chi-square, and Fisher's exact tests were used to determine if differences were statistically significant.

## 3. Results

Caries experience based on age and gender using two-way ANOVA was not statistically significantly different between individuals born with clefts and their relatives or unrelated unaffected individuals in all three study sites (Table 1). Females tended to show higher levels of caries experience than males.

## 4. Discussion

Our results do not support the suggestion that individuals born with isolated cleft lip with or without cleft palate have higher caries experience. Our study includes individuals from a wide range of ages and three geographically independent sites. In these sites, both individuals born with clefts and individuals born without clefts were derived from the community, and our study does not include a sample of convenient controls recruited from a hospital or composed by dental students.

The use of DMFT scores has its own limitations. This scoring system was created to be applied at 12 years of age. As one gets old, the DMFT score may increase not only due to new caries lesions, but also due to prosthetic work to replace missing units (which would increase the “F” component of the DMFT score, and tooth losses due to periodontal diseases or trauma, which would increase the “M” component of the DMFT score). Our study design minimizes this issue since the study subjects typically had no dental fillings and our protocol included questions related to history of dental trauma and reasons why teeth may have been extracted to avoid counting missing teeth extracted for reasons other than caries. Also, subjects included in this study were not exposed to any preventive dental measures and went to the dentist to address concerns related to dental pain, which was usually resolved through extractions.

A number of variables have been associated with oral clefts. These include seasonal variation, parental age, maternal age, birth order, emotional stress, other maternal risks, and socioeconomic status (reviewed by [37–39]). It is hard to exclude chance correlations, and a number of these findings have not been reproduced. The only demographic variable consistently associated with oral clefts is ethnicity. In comparison to Whites, Asians and American Indians have a higher frequency of oral clefts, whereas African and African descents have a lower frequency [40, 41]. Ethnicity can be a surrogate of lower socioeconomic status in some parts of the world, such as in the Americas, where Native Indians as an example tend to be disenfranchised [42]. However, the variation in frequency of oral clefts related to ethnicity also speaks in favor of a strong genetic component to the defect, which is likely influenced by many genes that can be modulated by the environment [43]. Lower socioeconomic status impacts access to care, and it has been suggested that children whose clefts had been surgically repaired have lower caries experience than those whose clefts had not been surgically repaired [18]. Since our study included families with lower socioeconomic status, our results are likely to be less influenced by the effects of inadvertently mixing individuals from different social strata. 

It continues to intrigue the finding that females have a higher caries experience than males. Lukacs and Largaespada [44] and Lukacs [45] propose three working hypotheses to explain this phenomenon.

- Female sex hormones and associated physiological factors can significantly affect cavity formation. Evidence from animal models suggests that female estrogens, but not male androgens, correlate with caries rates. It is possible that there is a cumulative effect of estrogens, including fluctuations at puberty and high levels during pregnancy, that promotes caries.
- Women produce less saliva than men do, reducing the removal of food residue from the teeth. During pregnancy, the chemical composition of saliva changes, reducing saliva's antimicrobial capacity.
- Women have food cravings, variations in immune response, and aversions during pregnancy. Women have an aversion to meat in the first trimester and crave high-energy, sweet foods during the third trimester.

In theory, if hormonal or physiological factors work independently or in an additive manner in women, their potential impact on the women's oral health can be significant. Our data supports the fact that caries experience in women increases in a more significant rate with age than in men, in diverse ethnic groups from different ecological and cultural settings, which supports the assumption that women are under additional influences that increase their caries rates.

X-linked genetic variation could partly explain why men tend to have lower caries rates than women. Our previous genome-wide linkage scan provided evidence of the involvement of the locus Xq27.1 in caries susceptibility [46]. A nonparametric LOD *P* value of  .0005 was found when the analysis considered individuals with lower caries experience rates. Our candidate gene approaches considering genes involved in enamel formation also suggested an involvement of chromosome X (with an association with markers in *amelogenin*, located at Xp22.3-p22.1) in caries susceptibility [47, 48].

In summary, our work provides strong evidence that individuals born with clefts are not more susceptible to caries. Women appear to experience higher caries rates throughout life and research should focus on understanding why gender appears to play a role in caries susceptibility.

## Figures and Tables

**Table 1:** Caries experience by age groups and gender in the studied populations.

	Cleft		Cleft		Cleft		Cleft		Cleft		Cleft		Unrelated		Unrelated		Unrelated	
	cases		cases		cases		relatives		relatives		relatives		individuas		individuas		individual	
	(Males)		(Females)		(Both)		(Males)		(Females)		(Both)		(Males)		(Females)		(Both)	
Age ranges (Years)	*N*	Average DMFT/ dmft	*N*	Average DMFT/ dmft	*N*	Average DMFT/ dmft	*N*	Average DMFT/ dmft	*N*	Average DMFT/ dmft	*N*	Average DMFT/ dmft	*N*	Average DMFT/ dmft	*N*	Average DMFT/ dmft	*N*	Average DMFT/ dmft
Philippines																		

Less than 6	4	7.2	1	0	5	5.8	12	4.5	9	4.8	21	4.7	0	—	0	—	0	—
6 to 12	21	9.4	21	8.9	42	9.2	25	6.8	30	7.2	55	8.1	0	—	2	1	2	1
13 to 18	28	7.1	23	11.3	51	8.8	30	6.1	31	6.9	61	6.2	3	1.3	7	5	10	3.9
19 to 25	19	12	8	7.9	27	10.8	25	7.4	19	7.7	44	7.6	3	6	13	6.9	16	6.8
26 to 35	16	9.7	4	13.5	20	10.4	18	8.3	25	8.4	43	8.3	3	3.3	10	4.9	13	4.5
36 to 44	5	16.4	1	30	6	18.7	26	7.7	29	13.4	55	10.7	1	6	10	7.4	11	7.3
45 or older	10	17.5	3	20	13	18.1	50	13.9	50	16.6	100	15.2	4	12.2	19	11.6	23	11.7

Total	103	11.3	71	13.1	174	11.7	186	7.8	193	9.3	379	8.7	14	5.8	61	6.1	75	5.9

Guatemala																		

Less than 6	15	0	8	0	23	0	4	0	3	0	7	0	30	1.2	23	1	53	1.1
6 to 12	7	2.4	2	0	9	1.9	6	0.2	1	0	7	0.1	38	2.7	34	4.2	72	3.4
13 to 18	8	2.8	3	6.3	11	3.7	3	2.7	7	2	10	2.2	16	1.8	29	2.8	45	2.4
19 to 25	0	—	1	11	1	11	5	3.8	13	5.6	18	5.1	29	2.4	61	4.4	90	3.8
26 to 35	0	—	0	—	0	—	5	6.4	9	7.3	14	7	24	4.7	57	7.3	81	6.5
36 to 44	0	—	0	—	0	—	4	9.8	7	5.6	11	7.1	17	8.9	37	14.1	54	12.4
45 or older	0	—	1	11	1	11	11	9.6	16	10.5	27	10.1	16	16.8	44	14.8	60	15.3

Total	30	1.7	15	5.7	45	5.5	38	4.6	56	4.4	94	4.5	170	5.5	285	6.9	455	6.4

Argentina																		

Less than 6	13	1.7	11	1.4	24	1.6	10	1.1	13	0.8	23	1	1	0	0	—	1	0
6 to 12	17	4.9	15	3	32	4	10	2.4	13	4.3	23	3.5	8	4.6	6	2.7	14	3.8
13 to 18	13	4.1	7	4	20	4.1	11	3.8	14	2	25	2.8	3	4.7	4	1.8	7	3
19 to 25	4	8.8	0	—	4	8.8	4	4	19	4.7	23	4.6	1	14	3	2.3	4	5.2
26 to 35	3	10	4	9.2	7	9.6	14	7.1	34	8.7	48	8.2	4	11.5	8	8.9	12	9.8
36 to 44	4	5.8	0	—	4	5.8	11	8.4	31	13.5	42	12.1	3	7	6	15.2	9	12.4
45 or older	2	13.5	4	18.5	6	16.8	11	15	29	16	40	15.8	1	10	2	13	3	12

Total	56	7	41	7.2	97	7.2	71	6	153	7.1	224	6.8	21	7.4	29	7.3	50	6.6

All Populations																		

Less than 6	32	2.97	20	0.47	52	2.47	26	1.87	25	1.87	51	1.9	31	0.6	23	1	54	0.55
6 to 12	45	5.57	38	3.97	83	5.03	41	3.13	44	3.83	85	3.9	46	3.65	42	2.63	88	2.73
13 to 18	49	4.67	33	7.2	82	5.53	44	4.2	52	3.63	96	3.73	22	2.6	40	3.2	62	3.1
19 to 25	23	10.4	9	9.45	32	10.2	34	5.07	51	6	85	5.77	33	7.47	77	4.53	110	5.27
26 to 35	19	9.85	8	11.35	27	10	37	7.27	68	8.13	105	7.83	31	6.5	75	7.03	106	6.93
36 to 44	9	11.1	1	30	10	12.25	41	8.63	67	10.83	108	9.97	21	7.3	53	12.23	74	10.7
45 or older	12	15.5	8	16.5	20	15.3	72	12.83	95	14.37	167	13.7	21	13	65	13.13	86	13

Total	189	6.67	127	8.67	316	8.13	295	6.13	402	6.93	697	6.67	205	6.23	375	6.77	580	6.3

Notes: Overall ANOVA *P* values: The Philippines (*P* = 0.14), Guatemala (*P* = 0.42), and Argentina (*P* = 0.63).
